# Infiltrative subependymoma of the brainstem: an uncommon presentation

**DOI:** 10.1590/0100-3984.2017.0186

**Published:** 2019

**Authors:** Bruno Niemeyer de Freitas Ribeiro, Rodrigo da Silva Mourão, Bernardo Carvalho Muniz, Nina Ventura

**Affiliations:** 1 Instituto Estadual do Cérebro Paulo Niemeyer, Rio de Janeiro, RJ, Brazil; 2 Universidade Federal do Rio de Janeiro (UFRJ), Rio de Janeiro, RJ, Brazil

**Figure 1:**
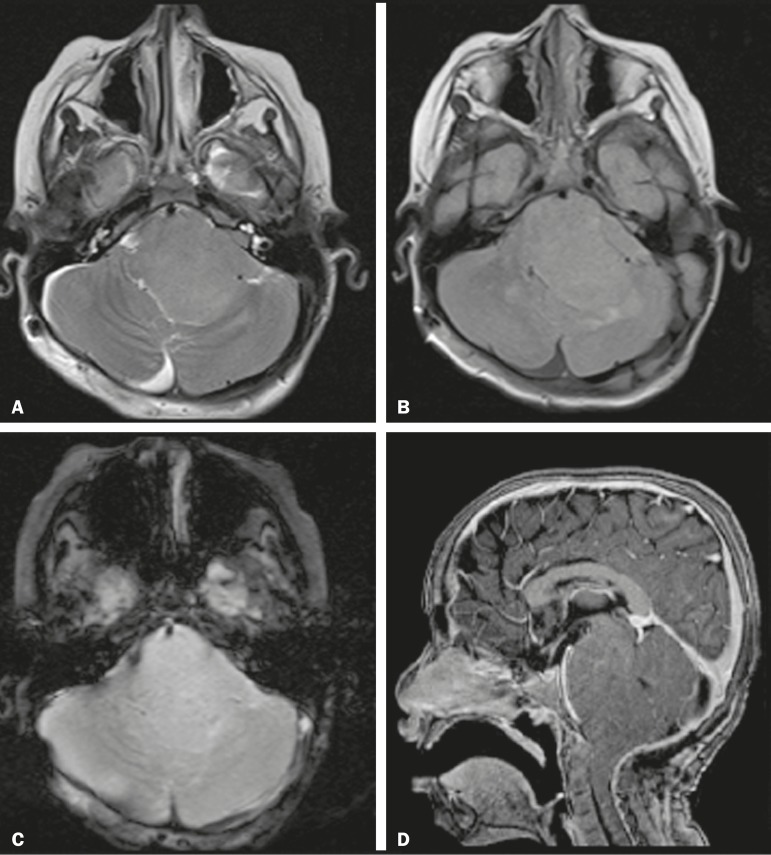
Magnetic resonance imaging. Axial T2-weighted sequence (**A**), axial T2-FLAIR sequence (**B**), axial T2*-weighted sequence (**C**), and contrast-enhanced sagittal T1-weighted sequence (**D**), showing an expansile, infiltrative lesion centered at the pontobulbar junction, with a slightly hyperintense signal in the T2-weighted sequences, although without cysts, calcifications, or contrast enhancement.

Dear Editor,

A 5-year-old male patient with a history of frequent falls and difficulty in speaking since two years of age presented with a two-month history of left peripheral facial paralysis, together with intractable vomiting and slurred speech. The mother reported that he had been born (at term) with perinatal asphyxia and had been admitted to the neonatal intensive care unit. Physical examination also showed left-sided paresis of the face, arm, and leg, as well as mild left-sided dysmetria. Magnetic resonance imaging of the brain showed a predominantly solid, infiltrative, expansile lesion in the brainstem, with a signal that was isointense on T1-weighted images and slightly hyperintense on T2-weighted images, without contrast enhancement or restricted diffusion (Figure 1). The lesion was resected, after which a diagnosis of subependymoma was made.

Subependymomas are tumors of uncertain origin, classified as grade I by the World Health Organization^(1)^. They typically occur in middle-aged or elderly individuals, having a slight predilection for males^(1-3)^. In 50-60% of cases, they are found in the fourth ventricle, although they can also arise in the lateral ventricles, third ventricle, and septum pellucidum, as well as in the cerebral, cerebellar, and medullary parenchyma^(1,2,4,5)^. Intracranial subependymomas are usually asymptomatic, generating symptoms only if they obstruct the flow of cerebrospinal fluid or compress the adjacent structures^(3)^. Histopathologically, they are characteristically fleshy, white, hypovascular, and well-defined^(3)^, composed of scattered groups of uniform cells, with cilia and microvilli, oval nuclei with little or no mitosis, and a matrix of dense fibrillar material, often accompanied by microcysts^(1,3)^.

When they occur in young individuals, subependymomas are usually mixed tumors, appearing in conjunction with ependymomas or astrocytomas^(4)^, and are typically infiltrative^(3)^. Among individuals with subependymoma who are under 14 years of age, the complete resection rates are lower and progression-free survival is shorter, especially when the lesion is infiltrative^(4)^, younger age and infiltration therefore being factors associated with a worse prognosis.

Expansile lesions in the nervous system have been the subject of recent studies in the radiology literature of Brazil^(6-10)^. On magnetic resonance imaging, they are typically solitary, circumscribed, solid-microcystic, intraventricular, and exophytic^(2,4,5)^. In comparison with the white matter, they present a signal that is isointense to hypointense on T1-weighted images and hyperintense on T2-weighted images, in some cases presenting hypointense foci in susceptibility-weighted sequences, due to calcifications or, more rarely, bleeding^(2,4,5)^.Such lesions typically show little or no contrast enhancement and rarely show any perilesional edema or restricted diffusion^(1,4,5)^. The main differential imaging diagnoses of intracranial subependymoma include ependymoma, medulloblastoma, astrocytoma, and central neurocytoma^(4)^, although diffuse glioma is the main differential diagnosis when the lesion is when infiltrative.

Surgical management is adopted only when a subependymoma is symptomatic, all other cases being monitored^(3)^. When the tumor is in a critical region, there is no need for extensive resection, given that even partial resection has been shown to result in a favorable outcome, especially if followed by radiosurgery and radiotherapy^(4)^. In conclusion, the case presented here was one of an unusual presentation of subependymoma, with characteristics indicative of a poorer prognosis. Such characteristics should be taken into consideration in imaging studies.
